# Liver/spleen magnetic resonance elastography and T1/T2 mapping in chronic liver disease: a prospective study

**DOI:** 10.1590/1806-9282.20241008

**Published:** 2024-12-02

**Authors:** Gökhan Mert Özyurt, Kaan Esen, Enver Üçbilek, Feramuz Demir Apaydın

**Affiliations:** 1 Mersin University, School of Medicine, Department of Radiology – Mersin, Turkey.; 2 Mersin University, School of Medicine, Department of Gastroenterology – Mersin, Turkey.

**Keywords:** Magnetic resonance, Liver cirrhosis, Magnetic resonance elastography

## Abstract

**OBJECTIVE:**

This study aims to compare liver and spleen stiffness measurements using magnetic resonance elastography with T1 and T2 relaxation times in patients with chronic liver disease.

**METHODS:**

A total of 75 chronic liver disease patients and 25 healthy volunteers underwent MR. Patients with significant liver fat and iron accumulation were excluded. Student’s t-test was employed to compare magnetic resonance elastography and T1/T2 values. Pearson’s correlation test was used to assess the relationship between magnetic resonance elastography and T1/T2 values.

**RESULTS:**

Liver magnetic resonance elastography showed a significant moderate positive correlation with liver T1 mapping (r=0.51, p<0.001) and liver T2 mapping (r=0.30, p=0.009) in patients. Spleen magnetic resonance elastography exhibited a significant moderate positive correlation with spleen T2 mapping (r=0.37, p=0.001). However, there was no significant correlation between spleen magnetic resonance elastography and spleen T1 mapping in patients. Spleen magnetic resonance elastography was moderately positively correlated with liver magnetic resonance elastography (r=0.30, p=0.01), and spleen volume showed positive correlations with spleen magnetic resonance elastography, spleen T1 mapping, and spleen T2 mapping. Cut-off values for liver magnetic resonance elastography, liver T1 mapping, and liver T2 mapping in patient and control groups were 2.6 kPa (AUC=0.97), 619 ms (AUC=0.90), and 52.5 ms (AUC=0.62), respectively.

**CONCLUSION:**

Relaxation methods offer noninvasive imaging without additional equipment. Liver T1 mapping may serve as an alternative to magnetic resonance elastography for chronic liver patient follow-up, while spleen T1 mapping is not reliable.

## INTRODUCTION

Understanding hepatic fibrosis in chronic liver disease is crucial in terms of disease prognosis and the follow-up of the patients^1^. While liver biopsy was previously considered the gold standard method for detecting liver fibrosis, recently, magnetic resonance elastography (MRE) has gained importance as a noninvasive method^2,3^. MRE offers advantages such as being noninvasive, reproducible, and reliable; however, it has certain limitations, such as the requirement of additional equipment and susceptibility to interference from iron and fat deposition^4^.

It has been reported that the measurement of T1 relaxation time can differentiate healthy subjects from patients with liver cirrhosis; however, it exhibits lower diagnostic performance compared to MRE^2,5^. T2 relaxation time can also differentiate the severity of fibrosis, and prolonged T2 relaxation time in regions of fibrosis may be associated with the coexistent inflammation and water content of the advanced fibrosis^2,6^. The main advantage of relaxation methods over MRE is that they do not require additional equipment. Liver iron deposition is a prominent technical limitation of both MRE and T1 relaxation measurements. In the liver with iron overload, MRE based on short echo time 2D spin echo echoplanar imaging has been utilized instead of gradient recalled echo MRE^7^. Similarly, the corrected T1 mapping technique that removes the effects of elevated iron from the T1 measurements has been shown to be closely correlated with liver biopsy and reliable for longitudinal monitoring of patients^8^.

In this study, we aimed to compare MRE and native T1\T2 relaxation times of liver and spleen in chronic liver disease patients and whether T1 and T2 relaxation times can be used as a noninvasive tool in the follow-up of patients.

## METHODS

This single-center prospective study was approved by the local ethics committee, and written informed consent was obtained from all participants. The study received support from the university’s scientific research projects unit.

Between May 2021 and January 2022, a total of 75 volunteer participants diagnosed with chronic liver disease and 25 healthy volunteers were included in the study. Among the healthy volunteers, 23 were males and 2 were females with an average age of 29.5 years (range 21–51 years). Among the patients, 42 were males and 33 were females with an average age of 62 years (range 30–85 years).

Volunteers who agreed to participate and had FIB-4>1.5 and/or APRI>0.5 were directed to MRE. Patients with liver stiffness>2.6 kPa were included in the study. Among the patients, 34 cases were associated with hepatitis B, 11 cases with hepatitis C, 16 cases were cryptogenic, 6 cases were associated with alcoholic cirrhosis, 2 cases were due to metabolic causes, 4 cases were related to primary biliary cholangitis, and 2 cases were associated with portal vein thrombosis.

Patients with pacemakers, implanted insulin or narcotic drug pumps, metallic heart valve prostheses, severe hepatic steatosis, iron accumulation, splenectomy, and a history of aneurysm treated with coils or malignancy were excluded from the study.

### MR protocol

Magnetic resonance images were acquired using a 1.5-Tesla MR system (Siemens Magnetom Aera; Siemens Medical Systems, Erlangen, Germany) equipped with a 32-channel body coil. An axial 3D multi-gradient-echo sequence with a multistep adaptive fitting algorithm reconstruction (Siemens LiverLab) was used to derive proton density fat fraction and R2* maps for assessing iron accumulation using T2* mapping. The parameters of the MR protocol are listed in Table 1.

**Table 1:** Parameters of MRI protocol.

	Sequences				
	2D EPI MRE	MOLLI 5(3)3	TrueFISP	T2* (T2 star) map	Multiecho GRE
TE (ms)	20	1.1	1.04	1,2,3,5,7,9,11,13,15	20
TR (ms)	50	277	190.39	200	15.6
FOV (mm^2^)	300x400	341x400	321x400	281x450	281x450
Slice thickness (mm)	10	8	8		10
Matrix	48x128	144x256	116x192	128x128	150x320
Flip angle		35	70	160	160
Distance factor		20%	20%		
Bandwidth (Hz/px)		1,085	1,184	1,080	710

2D EPI MRE: two-dimensional echoplanar imaging magnetic resonance elastography; MOLLI 5(3)3: 5(3)3 model Modified Look-Locker Inversion; GRE: gradient; TE: time echo; TR: time repetition; FOV: field of view.

For liver and spleen MRE, a two-dimensional (2D) echoplanar imaging (EPI) technique was employed. To perform the MRE, a passive driver (TelemedWave) was placed on the right upper abdomen for liver imaging and the left upper abdomen for spleen imaging, generating shear waves through continuous acoustic vibrations at a frequency of 60 Hz.

The 5(3)3 model Modified Look-Locker Inversion (MOLLI) recovery technique was used for liver and spleen T1 mapping, with the following inversion times (TI): 100, 180, 535, 895, 985, 1,220, 1,760, and 2,280 ms. For liver and spleen T2 mapping, the TrueFISP sequence was employed, which required three different preparation times (0, 25, and 55 ms).

### Image analysis

The image analysis was conducted using the syngo.via software provided by Siemens Healthcare, accessed through a server. The analysis was carried out independently by two readers, each with 4 and 20 years of experience in radiology, respectively. All images were taken at the same level of the liver. Liver MRE, T1 mapping, and T2 mapping measurements were performed by drawing geographic or ellipsoidal regions of interest (ROIs) from the widest possible area in a 95% confidence map, positioned 1 cm away from the portal vein and liver capsule^9^.

Axial T2-weighted images were loaded into the syngo.via workstation for spleen volume measurement, and a 3D image was generated through multi-planar reconstruction.

### Statistical analysis

All data were statistically analyzed using the Statistical Package for the Social Sciences program. A significance level of p<0.05 was set for statistical significance. Liver and spleen MRE, T1, and T2 values of healthy volunteers and patients were compared using the Student’s t-test, and the correlation between MRE, T1, and T2 values was assessed using the Pearson correlation test.

## RESULTS

When comparing the patient and control groups, statistically significant differences were observed in liver and spleen MRE, T1 mapping, and T2 mapping values (Table 2). The patient group exhibited higher values compared to the healthy group. The cut-off values for liver MRE, T1 mapping, and T2 mapping in differentiating between the patient and control groups were 2.6 kPa, 619 ms, and 52 ms, respectively (Table 3).

**Table 2:** Statistics of the patient and control group.

	Group	n	Mean	Standard deviation	p
Liver MRE	Control	25	1.9	0.3	0.000
	Patient	75	5.4	1.9	
Spleen MRE	Control	25	4.7	0.9	0.000
	Patient	74	9.6	3.2	
Liver T1 mapping	Control	25	578	33	0.000
	Patient	75	683	81	
Spleen T1 mapping	Control	25	1,118	46	0.000
	Patient	75	1,197	72	
Liver T2 mapping	Control	25	49	2	0.004
	Patient	75	51	4	
Spleen T2 mapping	Control	25	81	10	0.007
	Patient	75	89	13	

n: number; MRE: magnetic resonance elastography.

**Table 3:** ROC curve analysis of liver magnetic resonance elastography, T1, and T2 values.

	AUC	Cut-off	P	Sensitivity	Specificity
Liver MRE	0.970	>2.6 kPa	<0.0001	96 (88–99)	100 (86–100)
Liver T1 mapping	0.902	>619 ms	<0.0001	75 (63–84)	96 (80–99)
Liver T2 mapping	0.621	>52 ms	0.0372	33 (23–45)	100 (86–100)

AUC: area under the curve; MRE: magnetic resonance elastography; ROC: receiver operating characteristic.

In the comparison of the patient’s liver measurements, moderate positive correlations were observed between liver MRE and T1 mapping (r=0.51, p<0.001), liver MRE and T2 mapping (r=0.30, p=0.009), and liver T1 mapping and T2 mapping (r=0.29, p=0.011). A moderate positive correlation was found between spleen T1 mapping and T2 mapping (r=0.40, p<0.001). While a moderately positive correlation was observed between spleen MRE and spleen T2 mapping (r=0.37, p=0.001), no correlation was found between spleen MRE and spleen T1 mapping (p=0.333).

In the patient group, spleen MRE showed a positive moderate correlation with liver MRE (r=0.30, p=0.01). Although there was no correlation between spleen and liver T1 mapping (p=0.059), a positive correlation was detected between spleen and liver T2 mapping (r=0.49, p<0.001). Spleen volume showed a positive correlation with spleen MRE (r=0.48, p<0.001), spleen T1 mapping (r=0.30, p=0.01), and spleen T2 mapping (r=0.33, p=0.004).

A simple method widely used for the noninvasive prediction of liver fibrosis, the FIB-4 score, was calculated for all patients. The FIB-4 score was calculated using the formula: age (years) x AST (U/L)/{[PLT (10^9^/L) x ALT (U/L)]}^(1/2)10^. According to the FIB-4 score, 9 patients had mild, 20 had moderate, and 46 had advanced fibrosis.

## DISCUSSION

In the present study, among patients with chronic liver disease, liver T1 and T2 relaxation values obtained from T1 and T2 mapping demonstrated a moderate positive correlation with hepatic stiffness values measured by MRE. The pursuit of an accurate, reliable, noninvasive, and repeatable method for monitoring fibrosis in chronic liver disease is a significant goal for researchers. Relaxation methods and MRE are the primary noninvasive techniques for quantitatively assessing hepatic fibrosis^2^. In recent years, studies comparing the diagnostic performance of these methods taking into account different technical parameters and devices with varying magnetic field strengths have been reported. Ulmenstein et al. reported that T1 mapping exhibited lower diagnostic performance compared to MRE in diagnosing hepatic fibrosis^5^. Conversely, Breit et al. highlighted T1 mapping as a capable predictor for detecting liver fibrosis and cirrhosis^11^. The potential benefits of using T1 mapping with the administration of a contrast agent have also been explored in the literature. In a study conducted by Jin et al., extracellular volume calculated with gadopentetate dimeglumine contrast showed a strong correlation with liver fibrosis^12^. As mentioned earlier, the main objective of this study is to assess a repeatable technique for patient follow-up. In this regard, the possible side effects and cost-effectiveness of contrast agents may pose limitations.

A systematic review and meta-analysis reported that spleen stiffness may be more specific and accurate than liver stiffness for detecting portal hypertension^13^. The diagnostic performance of spleen stiffness was found to be similar to liver stiffness in predicting advanced liver fibrosis^14^. Talwalkar et al. noted a strong correlation between liver and splenic stiffness (r=0.75)^15^. In our study, spleen MRE exhibited a positive moderate correlation with liver MRE (r=0.30, p=0.01). However, spleen T1 relaxation did not show a correlation, either with spleen MRE (p=0.333) or with liver T1 relaxation (p=0.059). Obmann et al. mentioned a significant difference in spleen T1 relaxation times in patients with liver fibrosis, but the difference between patients with and without liver fibrosis was much smaller in the spleen than in the liver^16^. This might explain the discordance in the current study as the majority of our patients had advanced fibrosis.

While Cheng et al. found a positive correlation between the splenic volume and liver fibrosis stage, they also noted that the splenic volume was not sensitive in predicting early fibrosis stages^14^. However, in our study, the spleen volume did not exhibit a correlation with liver MRE. We believe this is because we did not classify patients according to the stage of fibrosis. Nevertheless, the spleen volume displayed a positive correlation with spleen MRE (r=0.48, p<0.001), spleen T1 mapping (r=0.30, p=0.01), and spleen T2 mapping (r=0.33, p=0.004).

Heye et al. reported T1 values for healthy subjects and patients with liver cirrhosis at 1.5T MRI as 678 ms and 852 ms, respectively^17^. Another study conducted with a 1.5T MRI device reported liver T1 relaxation times of 500±79 ms in normal patients, 574±84 ms in Child-Pugh A patients, and 690±147 ms in Child-Pugh B/C patients^18^. In the present study, the mean T1 value in the control and patient groups was 578 ms and 683 ms, respectively. It is important to note that the range of T1 values may vary due to differences in MR equipment and magnetic field inhomogeneity, underscoring the need to establish device-specific reference ranges. Additionally, it is crucial to consider the potential effects of acute liver diseases, hepatic iron overload, and steatosis on T1 mapping. Multiparametric MR imaging techniques that quantify hepatic iron and steatosis alongside mapping are recommended^5^.

It is essential to acknowledge the limitations of our study, including the preference of MRE as the reference method for liver fibrosis assessment instead of biopsy and the inability to calculate the extracellular volume due to the lack of contrast material. Additionally, while multiple elastogram maps from various levels are commonly obtained in the literature, our study conducted a single-level measurement.

## CONCLUSION

Liver T1 mapping holds promise for the follow-up of chronic liver disease, offering advantages like short examination times and no need for additional equipment. However, factors that may confound T1 relaxation times, such as acute liver diseases, hepatic iron overload, and steatosis must be considered. Furthermore, additional investigations are needed for the routine clinical use of spleen T1 mapping.
