# Erratum

**DOI:** 10.1590/1806-9282.20241008ERRATUM

**Published:** 2025-05-19

In the manuscript "Liver/spleen magnetic resonance elastography and T1/T2 mapping in chronic liver disease: a prospective study", https://doi.org/10.1590/1806-9282.20241008, published in the Rev Assoc Med Bras. 2024;70(12):e20241008, on page 1:

**Where it reads:**

Funding: none.

**It should read:**

Funding: This study was supported by the Mersin University Scientific Research Projects Coordination Unit under the project number 2021-1-TP3-4168.

